# Impact of Occupational Noise Exposure on Physical and Mental Health of Water Pumping Station Operators in Lebanon

**DOI:** 10.3390/ijerph23020262

**Published:** 2026-02-19

**Authors:** Rola Sammoura, Akram El Tannir

**Affiliations:** 1 Department of Industrial Engineering and Engineering Management, Faculty of Engineering, Beirut Arab University, Debbieh, Lebanon; 2 The Water Distribution Department, South Lebanon Water Establishment, Saida, Lebanon; 3 Department of Industrial and Mechanical Engineering, School of Engineering, Lebanese American University, Blat-Jbeil, Lebanon; akram.eltannir@lau.edu.lb

**Keywords:** noise, water sector, pumping stations, hearing impairment, mental health

## Abstract

**Highlights:**

**Public health relevance—How does this work relate to a public health issue?**

- Exposure to occupational noise adversely affects the physical and mental health of workers in the water sector, representing a significant public health concern.
- Workers in the public water sector in Lebanon constitute a vital part of the Lebanese workforce, responsible for the operation of an important public service. Their health protection and safety are essential and form an integral part of the overall public health protection.

**Public health significance—Why is this work of significance to public health?**

- This study is the first to assess the effect of occupational noise on workers in the Lebanese water sector, showing a gap in safety control measures and occupational health protection policies, and providing evidence for policymakers on occupational health.
- The findings of this study reveal adverse effects of noise on hearing, general stress level, sleep, and cognitive behavior, addressing the need for occupational health protection measures.

**Public health implications—What are the key implications or messages for practitioners, policymakers, and/or researchers in public health?**

- Lebanese policymakers can use the results to develop protective policies and implement noise reduction strategies to provide a safe working environment in water pumping stations.
- South Lebanon Water Establishment, with the collaboration of the Ministry of Public Health, can use the findings to implement a comprehensive monitoring program that includes regular audiometric testing and mental health screening for workers, supplemented by training and awareness programs to ensure their long-term safety.

**Abstract:**

This study investigates the impact of occupational noise on the physical and mental health of 50 water pumping station operators in Lebanon. The research aimed to quantify noise exposure, assess its effects on hearing and psychological well-being, and identify contributing factors. To achieve this, this study employed several evaluation methods. Noise exposure was measured using a calibrated sound level meter to determine the average A-weighted sound pressure levels (dBA) at 52 stations, which were then compared to the 85 dBA recommended limit from the National Institute for Occupational Safety and Health (NIOSH). Physical health, specifically hearing ability, was assessed using a validated smartphone-based pure-tone audiometry application to measure hearing thresholds across multiple frequencies. The resulting data were used to calculate the pure-tone average (PTA) and classify hearing impairment according to the World Health Organization (WHO) standards. Psychological health was evaluated through a structured 14-item questionnaire developed for this study, covering self-reported impacts on stress, anxiety, sleep quality, concentration, communication, and emotional state. The results indicated a hazardous work environment, with the mean noise level across stations (86.67 dBA) significantly exceeding the NIOSH safety threshold. A high prevalence of hearing impairment was observed among operators, with 88% exhibiting impairment in the worse ear. A multiple linear regression analysis revealed that noise level, age, and duration of exposure were all statistically significant predictors, collectively explaining 62.3% of the variance in hearing impairment (F(3, 46) = 25.32, *p* < 0.001). The analysis further identified age as a key effect modifier; the duration of exposure was the dominant risk factor for younger workers, while the intensity of the noise level was more critical for older workers. Psychologically, workers reported a high prevalence of adverse effects, with sleep disturbances being the most common issue (reported by 75%), followed by emotional distress (67%) and anxiety (60%). This study also found a complete lack of hearing protection use and no formal training on noise hazards, highlighting significant gaps in occupational safety practices.

## 1. Introduction

Noise is considered one of the most harmful physical factors affecting human physiological and psychological health [1]. In industrial settings, noise is a pervasive occupational hazard impacting worker health, safety, and productivity [2,3,4]. Extensive reviews consistently show that occupational noise exposure is a primary cause of preventable hearing loss and contributes to various other health problems, making its study and control essential for public health [5,6]. Accordingly, understanding the effects of noise on health becomes important in order to develop effective policies for prevention, regulation, and intervention.

The most direct and thoroughly documented consequence of continuous noise exposure is noise-induced hearing loss (NIHL), a progressive and permanent type of hearing impairment [5]. High rates of NIHL are observed in many high-risk industries. For example, a study in the steel industry found a substantial burden of hearing impairment among workers, highlighting the risks in heavy manufacturing [7]. The effects of NIHL go beyond just hearing difficulties; it is strongly linked to overall health problems, poor sleep quality, and significant workplace challenges, such as communication difficulties and increased safety risks [8].

A crucial aspect of NIHL is how it interacts with natural aging. Research indicates that noise exposure early in life can greatly speed up age-related hearing loss, suggesting that noise damage makes the auditory system more susceptible to age-related decline [9].

In addition to its adverse effect on hearing, occupational noise has a significant and widespread impact on the human body. The constant stress from a noisy environment can trigger various psychological and physiological responses. Studies have directly linked noise exposure to a higher occurrence of mental disorders and a reduced ability to work among industrial workers [10]. A systematic review of noise effects on seafarers identified numerous psychological issues, including anxiety, depression, and poor concentration [11]. This is supported by research showing that industrial noise, especially when combined with work-related stress, predicts poor psychological well-being [2].

Cognitive functions are also known to be affected. Noise interferes with complex mental processes, reducing concentration and worker productivity [12]. Physiologically, the body’s stress response to noise can lead to severe health consequences. A large study in U.S. industries found significant connections between workplace noise exposure and cardiovascular conditions, such as high blood pressure and elevated cholesterol [13]. Even low-frequency noise, often perceived as a vibration, has been shown to have harmful effects on physiological and mental health in controlled settings [14]. The overall result is a clear decline in workers’ general health and performance [3].

While the effects of industrial noise are widely studied, the specific source and context of the noise are crucial. For example, research on wind turbine noise has highlighted its impact on the general health of staff at wind farms [15]. Of particular relevance to the current study is the noise generated by diesel generators. A study conducted during the 2024–2025 electricity crisis in Ecuador found that the widespread use of diesel generators led to significant noise pollution, with low- and mid-frequency components linked to annoyance, sleep disturbance, and cardiovascular stress [16]. This directly parallels the situation in Lebanon’s water pumping stations, where reliance on generators creates a similarly hazardous acoustic environment.

While previous studies provide valuable insights into occupational noise exposure, they have mostly focused on industrial settings, leaving a gap in understanding unique operational contexts, such as water pumping stations, which are critical infrastructures in Lebanon. This research focuses on water pumping stations in Lebanon, specifically in the South and Nabatieh Governorates. These stations are managed by the South Lebanon Water Establishment (SLWE). The SLWE, headquartered in Saida, is a public authority responsible for the production, distribution, and maintenance of water systems in South Lebanon. The SLWE works under the umbrella of the Ministry of Energy and Water in Lebanon, and is responsible for providing safe and potable water to all cities, towns, and villages in South Lebanon [17]. Due to severe limitations in the national electricity supply from Electricité du Liban (EDL), the SLWE had to install diesel generators at most water pumping stations. These generators are operating particularly during power outages, leading to variable noise conditions. Thus, diesel generators ensure a continuous water supply but create substantial and continuous noise [16]. The accumulation of this noise, combined with the sounds of pumps, ventilation fans, boosters, and fluid turbulence within pipelines, creates an occupational environment where workers are exposed to noise for prolonged periods. This study aims to evaluate how noise exposure influences physical and psychological health outcomes among operators. Given the lack of region-specific studies, this project addresses a critical knowledge gap by providing quantitative and qualitative evidence on noise exposure and associated health risks while suggesting evidence-based recommendations for noise control and worker protection.

The objectives of this study are as follows:

- Measure and evaluate noise exposure levels at selected water pumping stations and classify them against occupational safety standards.
- Assess the physical (hearing ability) and psychological (stress, sleep disturbances, concentration, communication, and emotional state) health status of operators working in water stations.
- Analyze the relation between health status, sound levels, age, and duration of exposure to noise
- Propose recommendations and practical measures to minimize health risks and improve workplace safety.

## 2. Materials and Methods

### 2.1. Study Design

This is a cross-sectional observational study conducted to explore the relationship between occupational noise and physical and mental health outcomes among workers in water pumping stations in South Lebanon. The study was conducted from October to November 2025 at water pumping stations located in Saida, Jezzine, Zahrani, and Nabatieh districts of South Lebanon. These stations are operated and managed by the SLWE. To evaluate whether the water pumping system constitutes a noisy environment, data were collected from 52 water pumping stations through thirteen site visits. These stations are operating on the electric grid, supplemented by a daily average of 8 h of diesel generator operation. Each station is operated by a single operator.

This study was conducted and reported in accordance with the STROBE checklist for observational studies.

Ethical approval was obtained from the Beirut Arab University Institutional Review Board (IRB code: 2025-H-0002-E-R-0914 and 24 October 2025). Approval to collect data was also obtained and written informed consent was secured from the SLWE. All participants provided informed consent prior to participation in the study. Participants were informed of the study’s purpose, procedures, risks, and benefits, and were assured of the confidentiality of their data and the voluntary nature of their participation. This study was conducted in accordance with the principles of the Declaration of Helsinki and the ethical guidelines for research involving human subjects.

Supervisors from the SLWE accompanied the main author, who was the principal investigator, during all site visits to facilitate access to and identification of the stations. However, supervisors were not present during the surveys and hearing tests and did not constitute any bias to the collected data.

### 2.2. Participants

The target population of the study consists of workers employed as operators in water pumping stations managed by the SLWE in Saida, Jezzine, Zahrani, and Nabatieh districts. A key characteristic of the target stations was their power source, relying on the national electric grid supplemented by on-site diesel generators, as this hybrid operation is the primary source of significant occupational noise exposure. Stations with a continuous 24-h electricity supply from the grid or those operating on solar power were excluded from the target population, as they do not represent the same noise exposure environment.

The sampling strategy employed was a census of all eligible and accessible stations that met the power source criteria. A prior power analysis was not performed because the intention was to include the entire available population of interest rather than a random sample. The selection process proceeded as follows: an initial list of all SLWE stations in the target districts was reviewed, and only those operating with a generator-supplemented power grid were deemed eligible. During the data collection period, several of these eligible stations could not be included for logistical and safety reasons, such as (1) inaccessibility due to political instability in South Lebanon, (2) generators being non-operational due to a lack of fuel, making noise measurement impossible, and (3) generators being out of service for maintenance. This process resulted in a final list of 52 reachable and operational stations that were visited for data collection. During the site visits, two operators were unavailable, resulting in a final sample of 50 operators who participated in the hearing tests and survey.

This final sample size of 50 participants is considered adequate for the study’s planned multiple regression analysis. A widely cited rule of thumb suggests that for multiple regression, a minimum of 10 participants per predictor variable is recommended to ensure a stable model [18]. As our model includes three primary predictors (noise level, age, and years of exposure), the minimum required sample size would be 30 participants. Our sample of 50 comfortably exceeds this threshold, providing sufficient statistical power for the analysis.

Participants were eligible for inclusion if they were operators at one of the selected stations and had worked in their current role for at least one year. Individuals with a known history of congenital hearing loss were excluded. No participants met the exclusion criterion, and all 50 workers who were invited agreed to participate and were therefore included in the analysis.

### 2.3. Assessments and Measurements

#### 2.3.1. Noise Measurement

Sound pressure levels were measured using a Brüel & Kjær Type 2230 Sound Level Meter (Brüel & Kjær, Nærum, Denmark). Prior to the field measurements, the instrument was calibrated by a certified technician at the Beirut Arab University acoustics laboratory using a Brüel & Kjær Type 4230 sound level calibrator (94 dB at 1000 Hz). The measurement protocol was guided by the principles of ISO 9612:2025 regarding instrumentation and measurement conditions [19]. The study employed an area noise survey approach. The noise measurements were conducted by the main author. At each water pumping station, measurements were collected during normal operating hours (8:00 AM to 4:00 PM) when diesel generators were operational, as this represents the typical exposure scenario. To capture variability in noise exposure, A-weighted sound pressure levels (dBA) were measured at multiple locations within each station where operators perform their duties. These locations included the control rooms, the main pump area, the generator area, and designated rest areas. At each location, a 3-min average reading was recorded to account for temporal variability in noise levels. The entire noise assessment protocol at each station took approximately 15 min to complete.

The average sound level across all measured locations within a single station was then calculated. The justification for the average noise level is that the workers in the water pumping stations do not have a fixed, stationary location. Rather, workers move between different areas within each station based on operational requirements. The time spent at each specific location varies daily and is not predetermined. Given this operational reality, the average noise level per station is considered an appropriate measure for occupational noise exposure.

#### 2.3.2. Hearing Assessment

The hearing ability of each operator was assessed using the “Hearing Test” mobile application, a smartphone-based pure-tone audiometry tool, installed on a Techno Camon smartphone (Tecno Mobile, Shenzhen, China). Auditory stimuli were delivered via HAVIT H655BT headphones (HAVIT, Guangzhou, China). The device was calibrated using a standardized calibration procedure based on the hearing threshold of the normal-hearing person prior to the study, based on the instructions given by the application [20,21].

The smartphone-based hearing test has been validated in peer-reviewed studies and demonstrates strong agreement with clinical pure-tone audiometry [22,23]. A validation study comparing biologically calibrated mobile devices with standard PTA found a mean difference of 2.6 dB (95% CI 2.0–3.1), with 89% of results differing by 10 dB or less, and reported a sensitivity and specificity of 98% (95% CI 93–100%) and 79% (95% CI 71–87%), respectively [22]. More recent research examining smartphone-based hearing tests in 92 subjects found that smartphone tests accurately detected 97.5% of ears with hearing loss and 94.3% of ears with normal hearing. The accuracy demonstrated frequency-dependent variation, with detection rates of 100% at speech frequencies, 98.4% at higher frequencies, but only 73% at lower frequencies [23].

The pure-tone audiometry protocol, a standardized method for measuring hearing thresholds across various sound frequencies, was used. Hearing thresholds were evaluated at five frequencies: 500, 1000, 2000, 4000, and 8000 Hz [24]. For each frequency, the hearing threshold was determined using an adaptive procedure where the participant indicated whether they could hear a tone at progressively lower or higher intensities. Each participant underwent a single, complete hearing assessment. Tests were conducted for both the right and left ears. All hearing tests were conducted in a quiet room in the station, after operations were shut down, with ambient noise levels below 50 dBA as verified by the sound level meter. For stations where a sufficiently quiet room was not available, testing was conducted at a nearby quiet location. The approximate duration of each hearing test was 15–20 min.

All hearing assessments were performed by the main author, who served as the lead investigator for this study. To ensure procedural fidelity and standardization, the author underwent a period of self-training by thoroughly reviewing the official user manual and administration protocol provided by the developers of the “Hearing Test” application [21,22]. Prior to commencing data collection, the author conducted several pilot tests on non-study participants to ensure proficiency with the adaptive testing procedure and consistent application of the standardized protocol.

Hearing impairment was classified according to the World Health Organization (WHO) standards as Normal (<20 dB HL), Mild (20–34 dB HL), Moderate (35–49 dB HL), Moderately severe (50–64 dB HL), Severe (65–69 dB HL), or Profound (≥80 dB HL) for both the right and left ears and the corresponding hearing impairments in decibels Hearing Level (dB HL), calculated as the average threshold at 500, 1000, 2000, and 4000 Hz, were also recorded [24].

#### 2.3.3. Psychological Health Assessment

To assess the effect of noise on the mental health of station operators, a psychological binary-response survey was used. The questionnaire was developed by the authors based on a comprehensive review of the literature on the psychological effects of occupational noise exposure [1,8,11]. It addresses six aspects identified in prior research: stress, anxiety, sleep quality, concentration, communication, and emotional state. The questionnaire comprises fourteen questions, with workers’ responses categorized into two groups: “Yes” and “No.” Data were collected by the main author through structured face-to-face interviews with workers. Neutral language was used to avoid leading questions. Table 1 presents the questionnaire along with the objective of each question as it relates to the study.

### 2.4. Statistical Analysis

Data analyses were performed using SPSS16.0 to obtain descriptive and inferential statistics. These analyses were performed to examine the effects of sound pressure levels on hearing, subject to three main factors—sound level, exposure to noise, and age—and mental health. The statistical analyses address the following:

- Hypothesis-testing for noise level assessment: A one-sample *t*-test was conducted to test whether the average noise level in a particular water pumping station, during diesel generator operation, significantly exceeds 85 dBA. The National Institute for Occupational Safety and Health (NIOSH) recommends an occupational noise exposure limit of 85 dBA over an eight-hour workday with a 3 dBA exchange rate to minimize the risk of hearing loss [25], a threshold also reflected in the WHO guidance and the European Union (EU)’s more protective regulatory framework [26,27]. In contrast, the U.S. Occupational Safety and Health Administration (OSHA) permits a higher exposure limit of 90 dBA over eight hours using a 5 dBA exchange rate, indicating less stringent protection [28]. Table 2 provides a comparative summary of the OSHA, NIOSH, WHO, and EU standards and guidelines. The 85 dBA and 90 dBA thresholds are widely used as benchmarks in occupational noise research [8,29,30]. The NIOSH limits, being more conservative and health protective, will be used as the threshold for this study. Although an average sound level was calculated for each station, the 85 dBA threshold will be used as the benchmark for assessing noise exposure in this environment.

The objective of this statistical evaluation was to determine whether the average noise level within the working environment of water pumping stations in South Lebanon exceeds the 85 dBA limit, thus justifying the implementation of a comprehensive noise control protocol. Null hypothesis: μ = 85 dBA; Alternative hypothesis: μ > 85 dBA; Significance level: α = 0.05 (one-tailed).

- Regression Model-Hearing Impairment Assessment: Linear regression analysis was conducted to examine the relationship between exposure to noise, age, and noise level as independent variables and hearing impairment as the dependent variable. Model assumptions were tested: normality of residuals (Shapiro–Wilk test, *p* > 0.05), homogeneity of variance (visual inspection of residual plots), independence of observations (Durbin–Watson statistic, 1.5–2.5), and multicollinearity (variance inflation factor, VIF < 2.5). Model fit was assessed using R^2^ and F-statistic at a 5% significance level. The primary analysis focused on the worse-ear PTA hearing impairment metric, incorporating the three independent variables and then stratifying the model by age groups above and below the mean. As the primary objective of this study is to determine the hearing damage resulting from noise exposure, the maximum hearing impairment (worse-ear impairment) has been used in the subsequent analysis. This methodology ensures that the results reflect the extent of hearing loss and capture the maximum degree of occupational hearing loss experienced. A sensitivity analysis for the better-ear PTA and binaural average metrics is provided in Appendix A.
- Psychological Assessment: Psychological health impacts of noise exposure were assessed using the 14-item binary questionnaire developed for this study. As this instrument has not been validated, analysis was limited to descriptive statistics, reporting the prevalence of endorsement for each item.

## 3. Results

### 3.1. Noise Level Assessment

The first objective of this study involves testing whether the environment at the water pumping stations, particularly those operating on a hybrid system of the national grid and diesel generators, constitutes a noisy environment during generator operation. Accordingly, the first question of this study asks whether the overall occupational environment in water pumping stations in South Lebanon constitutes a noisy, critical environment that exceeds occupational safety standards. This is a policy-relevant question, as it informs whether system-wide interventions, such as hearing conservation programs and engineering noise controls, should be implemented across the water utility.

**Figure 1:**
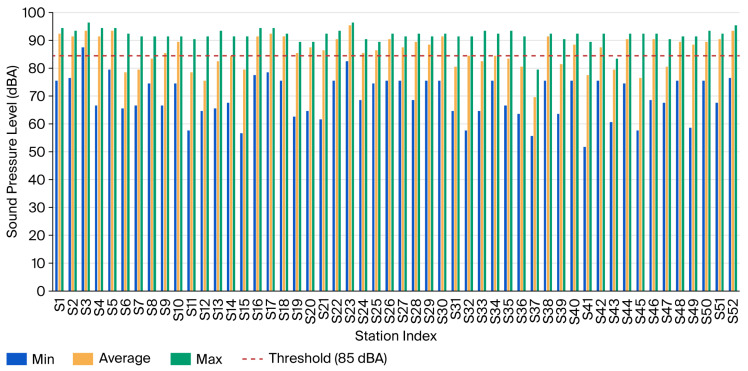
The max., average, and min. sound pressure levels and NIOSH threshold of 85 dBA.

Sound pressure levels (SPLs) measured at each water station are shown in Figure 1. There is heterogeneity in noise levels across stations, with some stations having average levels below 85 dBA and others well above. Specifically, 68% of stations (*n* = 35) had average noise levels equal or exceeding 85 dBA (95% CI: 55.32–80.67%), while 32% of stations (*n* = 17) had average levels below this threshold. The range of the average noise level across stations was 70 to 96 dBA.

A one-sample *t*-test was conducted to test whether the average noise level in water pumping stations significantly differs from 85 dBA. The results show that the average noise level (M = 86.67 dBA, SD = 5.67) significantly exceeds the 85 dBA threshold (t-statistic (51) = 2.127, *p* = 0.019, one-tailed). This finding indicates that the overall working environment in water pumping stations in South Lebanon exceeds occupational safety standards, justifying the implementation of a comprehensive noise control protocol applicable across the utility.

### 3.2. Physical and Psychological Health Assessment

The second objective of this study is to investigate the effect of exposure to noise on the health of operators working in water pumping stations. Demographic information for all 50 workers was collected using three questions, as summarized in Table 3.

The mean age of the participants was 52.36 years (SD = 9.79, Range: 27–70). The majority of workers (58%) were between 50 and 60 years of age. Workers above 60 constituted 20% of the sample, while those under 50 represented 22%. In terms of work experience, workers reported a mean year of experience of 16 years (SD = 9.53, Range:1–44), and 70% of the participants have been employed in this environment for more than 10 years. Among these, 2.86% are under the age of 40, 71.42% are between 40 and 60 years old inclusive, and 25.71% are over 60.

#### 3.2.1. Physical Health Assessment: Hearing Conditions

Hearing assessments were conducted for all 50 participants. The mean hearing impairment was 28.58 dB HL (SD = 11.08) in the better-ear and, as expected, was higher in the worse-hearing ear at 34.92 dB HL (SD = 12.06). Individual thresholds ranged from 10.00 to 56.00 dB HL for the better ear and 14.00 to 65.00 dB HL for the worse ear. The prevalence of hearing impairment, defined as a pure-tone average (PTA) ≥ 20 dB HL, was 76% (*n* = 38) in the better ear and 88% (*n* = 44) in the worse ear. In the better ear, 24% of participants had normal hearing, while 46% exhibited mild impairment, 24% moderate impairment, and 6% moderately severe impairment, with no cases of severe impairment observed. In contrast, only 12% of participants demonstrated normal hearing in the worse ear, with impairment severity distributed as mild (36%), moderate (40%), moderately severe (10%), and severe (2%). Unilateral hearing loss, defined according to WHO criteria as normal hearing in the better ear (PTA < 20 dB HL) and impaired hearing in the worse ear (PTA ≥ 35 dB HL), was uncommon in this sample and was identified in only 2 of the 50 participants [31].

**Figure 2:**
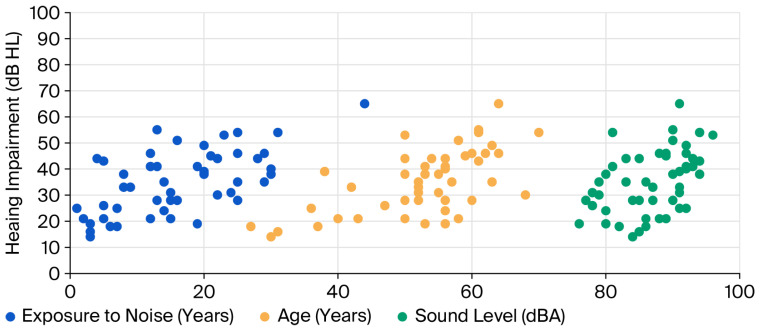
Plot of the hearing impairment with sound level, age, and exposure to noise.

To quantify the regression relationship between hearing impairment (as the dependent variable) and three independent variables: sound level, noise exposure, and age, a multiple linear regression model was developed. Figure 2 presents scatter plots illustrating the relationship between the three independent variables on the horizontal axis and the dependent variable on the vertical axis. The plot shows that an increase in any of the three independent variables is associated with an increase in hearing impairment.

The results of the regression analysis are displayed in Table 4. The results demonstrate the usefulness of the model, with an R^2^ value of 0.623, indicating that approximately 62.3% of the variability in the hearing impairment can be explained by sound level, age, and exposure to noise. The overall regression model is statistically significant (F(3, 46) = 25.32, *p* < 0.001), indicating that the overall model appears to be statistically useful for predicting hearing impairment.

The average sound level has a positive and statistically significant effect on the hearing impairment (*p* < 0.001). For every additional dBA in sound level, hearing impairment was higher by approximately 0.772 dB HL, assuming age and exposure to noise remain constant. Age also shows a strong positive and significant effect (*p* < 0.001). Each additional year of age corresponds to an increase of about 0.571 dB HL in the hearing impairment, controlling for sound level and exposure to noise. Exposure to noise is also significant. For each additional dBA increase, the hearing impairment increases by 0.366 dBA HL, holding sound level and age constant. The intercept is statistically significant but is not meaningful alone. Thus, all predictors are statistically significant at the 0.05 level. The confidence intervals do not include zero, which supports the significance.

An analysis of the model residuals was conducted to verify the underlying statistical assumptions. The results confirmed the normality of residuals (Shapiro–Wilk test, *W* = 0.978, *p* = 0.465), the independence of observations (Durbin–Watson statistic = 2.11). Homoscedasticity was assessed by visual inspection of the scatterplot of residuals against predicted values. The plot revealed no systematic pattern, indicating that the assumption of constant variance was satisfied. Furthermore, the absence of multicollinearity was established, with all variance inflation factors (VIFs) well below the commonly accepted threshold of 5 (VIF range: 1.04–1.54), validating the robustness of the model.

A sensitivity analysis was conducted to assess the robustness of the developed regression model. Each covariate was excluded individually to evaluate its influence on the overall model. When years of noise exposure were excluded, both sound level (β = 0.873, *p* < 0.001) and age (β = 0.773, *p* < 0.001) remained strong predictors, with the model explaining 56.88% of the variance. Excluding age revealed that sound level (β = 0.687, *p* = 0.004) and noise exposure (β = 0.708, *p* < 0.001) remained significant, accounting for 47.97% of the variance. When sound level was excluded, age (β = 0.514, *p* = 0.001) and noise exposure (β = 0.477, *p* = 0.004) continued to be significant predictors, with 49.61% of the variance explained. The results remain robust. Age, sound level, and exposure to noise consistently appear as strong predictors.

Results of the sensitivity analysis using alternative hearing impairment metrics (better-ear and binaural average thresholds) are presented in Appendix A. When using the binaural average hearing threshold as the outcome, the model was statistically significant (F(3, 46) = 18.38, *p* < 0.001), explaining 54.5% of the variance. All three predictors were significant: sound level (β = 0.624, *p* = 0.002), Age (β = 0.523, *p* < 0.001), and exposure to noise (β = 0.297, *p* = 0.044). For the better-ear threshold, the model remained statistically significant (F(3, 46) = 9.79, *p* < 0.001), explaining 38.9% of the variance. In this model, sound level (β = 0.478, *p* = 0.043) and age (β = 0.476, *p* = 0.005) were significant predictors, whereas exposure to noise was not (*p* = 0.173). The most sensitive indicator of occupational damage is the hearing threshold in the worse ear. Although age is a major confounder, the analysis accounts for its effect and demonstrates that both the sound level and, importantly, the cumulative duration of noise exposure are significant contributors to hearing impairment in this group of workers.

Stratified analyses by age group showed that the relationship between noise exposure, noise level, and hearing impairment was present in both younger (<53 years) and older (≥53 years) workers. The results are displayed in Table 5.

In the younger group, the model was significant (F(2, 18) = 16.714, *p <* 0.001) and explained 65% of the variance. Years of exposure to noise was a very strong predictor (β = 0.911, *p* < 0.001), while sound level was not significant (*p* = 0.058). In the older group, the model was also significant (F(2, 26) = 7.295, *p* = 0.003) but explained less variance (R^2^ = 0.359). Sound level was a significant predictor (β = 0.932, *p* = 0.01), while exposure to noise was not significant (*p* = 0.068). There is evidence of effect modification by age. For younger workers, years of exposure is the dominant factor. For older individuals, noise level is more important, likely because age-related hearing loss is already a major factor. These findings highlight the need for age-specific hearing conservation strategies. For younger workers, interventions should prioritize reducing cumulative exposure duration, while for older workers, emphasis should be placed on engineering controls to lower noise intensity.

#### 3.2.2. Psychological Health Assessment

To assess the impact of noise exposure on workers’ mental health, a binary (Yes/No) questionnaire-based survey was conducted (refer to Table 1). The results are presented in Table 6.

Descriptive analysis of the psychological questionnaire indicated a high prevalence of self-reported symptoms among noise-exposed workers. The findings reveal that, on average, more than 50% of respondents reported noise-related issues such as stress (55%), anxiety (60%), sleep disturbances (75%), concentration difficulties (53%), impaired communication (57%), and emotional problems (67%). Sleep-related symptoms were the most frequently reported, with 70–80% of workers endorsing items related to sleep disturbance and broader health effects of workplace noise. Emotional symptoms were also common, reported by 66–68% of workers, while stress-related symptoms showed moderate prevalence, ranging from 48% to 64% across items. Anxiety related to workplace noise was reported by 60% of workers. Concentration-related outcomes were more variable, with endorsement ranging from 32% for general concentration difficulties to 70% for cognitive or intellectual effects, which reflects the effect of noise exposure on work performance necessitating repetition or revision of work. Communication-related difficulties were reported by 56–58% of workers. Overall, these findings suggest that sleep disturbance, cognitive impact, and emotional strain are the most prominent self-reported psychological concerns in this occupational setting, while stress and effects on concentration vary across specific aspects, highlighting the multifaceted and exploratory nature of psychological responses to occupational noise exposure.

### 3.3. Awareness

In addition to the previously analyzed objectives, this study also explored the awareness of workers regarding health risks associated with prolonged noise exposure, along with the importance of taking appropriate precautions.

**Figure 3:**
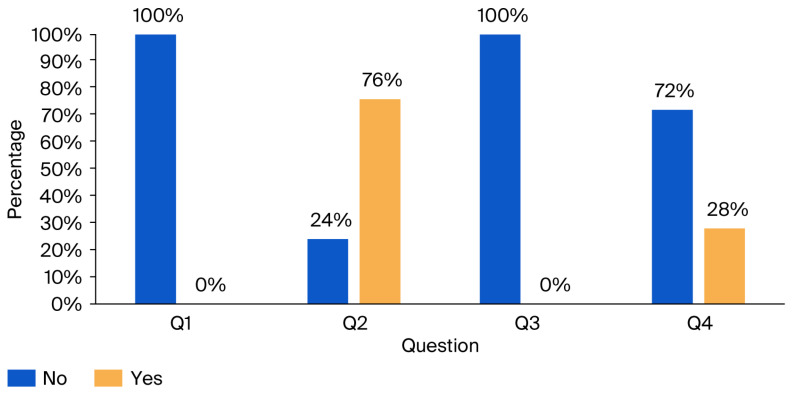
Results of the noise hazards and protective measures awareness questionnaire: percentage of “No” and “Yes” responses.

This section comprises four questions aimed at evaluating workers’ awareness of the health impacts of noise exposure and the importance of using protective equipment in high-noise environments. The questions are presented in Table 7, and the corresponding results are illustrated in Figure 3.

The findings reveal that operators do not use any hearing protection at work, and 76% recognize the potential health hazards associated with prolonged exposure to high noise levels. No training has been provided regarding the impact of noise on health and the necessity of using hearing protection. Merely 18% of participants have undergone a hearing examination conducted by a professional physician.

## 4. Discussion

This study presents the first comprehensive assessment of occupational noise exposure and its associated physical and psychological health effects among water pumping station operators in Lebanon. The findings demonstrate a clear association between noise exposure and adverse health outcomes, alongside substantial deficiencies in occupational noise control practices. The mean noise level across the 52 stations was 86.67 dBA, exceeding the National Institute for Occupational Safety and Health (NIOSH) recommended exposure limit of 85 dBA [25]. Notably, 68% of the stations met or exceeded this threshold, highlighting the urgent need for targeted interventions. These results are consistent with those found in other industrial settings, in Ecuador during the 2024–2025 electricity crisis, where the reliance on diesel generators dramatically increased environmental noise pollution and associated health risks [16].

The physical health assessments revealed a high prevalence of hearing impairment among workers, with 88% demonstrating impaired hearing (PTA ≥ 20 dB HL). This prevalence is comparable to or higher than that reported in other noise-intensive industries, such as steel manufacturing (47% in Egypt) and general manufacturing (34.75% in Iran) [5,7]. Regression analysis identified noise level, age, and years of exposure to noise as significant predictors of hearing impairment, jointly explaining 62.3% of the observed variance. These findings are consistent with established evidence on the determinants of noise-induced hearing loss [6]. The loss of statistical significance for “exposure to noise” in the better-ear does not reflect a weakness in the data. Rather, it reflects the asymmetric nature of noise-induced hearing loss and the dominant, universal influence of age-related hearing decline. Because the better-ear metric captures the least affected ear, it tends to attenuate the observable impact of occupational noise, while the effect of aging remains strong across both ears [32].

Age emerged as an important effect modifier in the relationship between noise and hearing loss. Among younger workers, the duration of exposure to noise was the dominant predictor, whereas noise level was more significant in older workers. This pattern suggests an interaction between occupational noise exposure and age-related hearing loss. The hearing system of older workers, already compromised by aging, appears to be more vulnerable to damage from noise levels. This finding is supported by research showing that ears exposed to noise age faster than unexposed ears. When workers experience noise damage early in their careers, it can speed up age-related hearing loss. This suggests that older workers whose hearing has already been damaged by noise are more vulnerable to further hearing loss as they continue to age and remain exposed to workplace noise [9].

In addition to the hearing assessment, the study identified a high prevalence of self-reported psychological symptoms among noise-exposed workers, consistent with prior research on occupational noise. Sleep disturbance was the most frequently reported issue, affecting 75% of workers, followed by adverse cognitive performance (70%), emotional distress (67%), general stress (64%), and anxiety (60%). These findings are in agreement with existing research that links chronic noise exposure to a range of adverse psychological outcomes, including cognitive decline, increased stress, and sleep disruption, which in turn can elevate the risk for cardiovascular conditions [2,5,8,10,11,12]. On the other hand, the prevalence of communication difficulties observed in the study averaged 57%, which is notably lower than the levels reported in other studies, where communication impairment has been shown to reach 63.3% [4]. This is supported by qualitative feedback, which identified that workers have their conversations outside or away from the pumping station and have become accustomed to the noisy environment.

The findings of this study should be interpreted in the context of several limitations. First, the cross-sectional design inherently limits the ability to establish causal relationships between noise exposure and hearing impairment, although the observed exposed-outcome associations are consistent with such a link. While the developed models controlled for key demographic and occupational variables, the potential omission of important confounders and unmeasured factors could have influenced the results. Given the nature of the work and the fact that all operators are exposed to some level of noise, the study also lacks a control group, which limits the ability to establish a causal relationship between noise exposure and hearing outcomes. Second, regarding our measurement protocol, the audiometric data were collected using a validated smartphone-based application. Although this approach enhances the feasibility and accessibility of hearing assessment in field settings, particularly in resource-constrained environments, it is not a substitute for clinical pure-tone audiometry for diagnostic purposes. Finally, the psychological outcomes were assessed with a non-validated, self-developed questionnaire. Consequently, these findings should be considered as exploratory and require further investigation with standardized psychometric instruments. Future longitudinal studies are needed to confirm these findings and to better understand how age and noise exposure contribute to hearing loss over time.

Despite these limitations, the findings suggest that occupational noise exposure in water pumping stations poses a health risk to workers. Evidence-based interventions should be implemented to reduce noise exposure and protect workers’ health. These interventions may include

1. Implementing engineering noise controls (e.g., silencers, enclosures);
2. Enforcing mandatory use of hearing protection devices (HPDs);
3. Conducting regular health monitoring and awareness campaigns;
4. Provide regular audiometric testing and mental health screening;
5. Develop occupational health policies at the institutional and governmental levels;
6. Integrating noise control in station design and operations;
7. Adopting administrative measures such as rotating workers to minimize their exposure to noise.

## 5. Conclusions

A study was conducted to assess the impacts of occupational noise on human well-being in water pumping stations in Lebanon. The results indicate that water pumping stations located in the South and Nabatieh Governorates are characterized by elevated noise levels and a high prevalence of adverse physical and psychological health indicators among workers. Based on benchmark exceedances and observed health indicators, precautionary evidence-based interventions should be implemented. Future research should incorporate appropriate comparison groups, longitudinal study designs, and validated instruments for psychological health assessment to better evaluate potential causal relationships, as the present study is limited to identifying observed associations rather than causation.

## Figures and Tables

**Table 1:** Psychological health questionnaire.

Category		Question	Aim
Stress	Q1	Would you say exposure to noise increases your stress levels?	General assessment of the relationship between noise exposure and stress levels.
	Q2	Do you feel more stressed after spending time in a noisy environment?	Short-term effect of being in a noisy environment
	Q3	Do you notice physical reactions in your body (e.g., increased heart rate, tension) during moments of high noise exposure?	Assess the long-term impact of noise on stress levels
Anxiety	Q4	Do you feel worried in no time due to workplace noise?	Direct impact of workplace noise on feelings of worry
Sleep Quality	Q5	Have you experienced sleep disturbance since working here?	Experience with sleep disturbances related to the work environment.
	Q6	Does noise in your work environment affect your ability to sleep well at night?	Explicitly links noise in the work environment to sleep quality
	Q7	Is it easy for you to fall asleep in a completely silent environment?	Highlight the broader impact of workplace noise on health.
Concentration	Q8	Would you say noise affects your ability to concentrate in your tasks?	General effect of noise on concentration.
	Q9	Do you find it harder to complete tasks accurately when there is noise in your work environment?	Address the quality of work done.
	Q10	Do you often find yourself needing to revise information or repeat steps due to distractions from noise in your environment?	Cognitive/Intellectual effects of noise.
Communication	Q11	Does workplace noise affect your ability to communicate with colleagues?	Overall impact of noise on communication
	Q12	Does noise make it difficult for you to hear and understand conversations at work?	Listening challenges posed by noise
Emotional State	Q13	Do you often feel irritated after being in a noisy setting?	Immediate emotional responses
	Q14	Have you noticed any prolonged mood fluctuations that you associate with noise exposure?	Long-term impacts of noise on mood.

**Table 2:** Comparative summary of NIOSH, OSHA, WHO, and EU noise exposure standards.

Feature	OSHA	NIOSH	WHO	EU
Legal Status	Enforceable federal regulation	Recommendation	Guidance	Binding directive
Exposure Limit	90 dBA (8 h Time-Weighted Average (TWA) with action level at 85 dBA for hearing conservation	85 dBA (8 h TWA)	Methodologies for disease burden (85–90 dBA, >90 dBA) Community guidelines include 70 dBA (24 h) for industrial areas	87 dBA with upper action level at 85 dBA and lower action level at 80 dBA
Exchange Rate	5 dB	3 dB		3 dB

**Table 3:** Demographic profile of operators.

Question	Aim
How old are you?	To collect the age of each worker.
How long have you been working in this environment?	To collect data concerning the job experience.
How many hours per day do you spend in the water pumping station?	To determine the number of hours per day each worker works in the station.

**Table 4:** Worse-ear hearing threshold regression model summary.

**R**	**R^2^**	**Adjusted R^2^**	**Std. Error of the Estimate**			
0.789	0.623	0.598	7.64			
F-Statistic, *p*-Value	F(3, 46) = 25.326, *p*-value < 0.001					
**Predictor**	**B**	**SE**	**β**	**t-** **Statistic**	***p***	**95% CI**
Average sound level (dBA)	0.772	0.196	0.363	3.932	<0.001	(0.38, 1.17)
Age (years)	0.571	0.137	0.463	4.178	<0.001	(0.29, 0.85)
Exposure to noise (years)	0.366	0.142	0.289	2.569	0.014	(0.08, 0.65)
Constant	−67.752	18.396		−3.683	<0.001	(−104.78, −30.72)

B = unstandardized coefficient; β = standardized coefficient; SE = standard error; *p*-value (two-tailed).

**Table 5:** Regression model summary of hearing threshold by age group.

**Younger Group (Age < 53 Years, *n* = 21)**					
**R**	**R^2^**	**Adjusted R^2^**		**Std. Error of the Estimate**	
0.806	0.65	0.611		6.105	
F-Statistic, *p*-Value	F(2, 18) = 16.714, *p*-value < 0.001				
**Predictor**	**B**	**SE**	**β**	**t-Statistic**	***p***
Average sound level (dBA)	0.459	0.227	0.282	2.024	0.058
Exposure to noise (years)	0.911	0.169	0.751	5.383	<0.001
Constant	−21.781	19.626		−1.11	0.282
**Older Group (Age ≥ 53 Years, *n* = 29)**					
**R**	**R^2^**	**Adjusted R^2^**		**Std. Error of the Estimate**	
0.599	0.359	0.310		9.475	
F-Statistic, *p*-Value	F(2, 26) = 7.295, *p*-value = 0.003				
**Predictor**	**B**	**SE**	**β**	**t-Statistic**	***p***
Average sound level (dBA)	0.932	0.337	0.447	2.768	0.01
Exposure to noise (years)	0.373	0.196	0.307	1.903	0.068
Constant	−48.644	28.728		−1.693	0.102

B = unstandardized coefficient; β = standardized coefficient; SE = standard error; *p*-value (two-tailed).

**Table 6:** Results of mental health surveys.

Category	Question	Proportion of Workers Saying “Yes”	Average per Category
Stress	Q1	0.64	0.55
	Q2	0.48	
	Q3	0.54	
Anxiety	Q4	0.6	0.60
Sleep Disturbance	Q5	0.74	0.75
	Q6	0.7	
	Q7	0.8	
Concentration	Q8	0.32	0.53
	Q9	0.56	
	Q10	0.7	
Communication	Q11	0.56	0.57
	Q12	0.58	
Emotional Status	Q13	0.68	0.67
	Q14	0.66	

**Table 7:** Noise hazards and protective measures awareness questionnaire.

	Question	Aim
Q1	Do you use any form of hearing protection in noisy areas of your workplace?	To gauge the use of hearing protection.
Q2	Are you aware of the potential health risks associated with prolonged exposure to loud noise?	To assess baseline awareness of noise-related health risks
Q3	Has there been training, or information provided about noise and hearing protection?	To determine awareness and educational efforts regarding noise hazards.
Q4	Have you had your hearing tested by a professional?	To check for proactive health monitoring related to noise exposure.

## Data Availability

Data collected and analysis scripts for this study are available from the corresponding author upon request.

## Abbreviations

The following abbreviations are used in this manuscript:

NIHL	Noise-induced hearing loss
SLWE	South Lebanon Water Establishment
EDL	Electricity du Liban
dBA	A-weighted decibels
WHO	World Health Organization
dB HL	Decibels hearing level
NIOSH	The National Institute for Occupational Safety and Health
EU	The European Union
OSHA	The Occupational Safety and Health Administration
TWA	Time-weighted average
SPL	Sound pressure level
PTA	Pure-tone average

## Appendix A

This appendix includes the results of the regression models using the binaural average hearing threshold and the better-ear threshold.

**Table A1:** Binaural average hearing threshold model summary.

**R**	**R^2^**	**Adjusted R^2^**	**Std. Error of the Estimate**			
0.738	0.545	0.515	7.738			
F-Statistic, *p*-Value	F = 18.384, *p*-value < 0.001					
**Predictor**	**B**	**SE**	**β**	**t** **-Statistic**	***p***	**95% CI**
Average sound level (dBA)	0.624	0.198	0.319	3.144	0.002	(0.23, 1.03)
Age (years)	0.523	0.138	3.786	3.786	<0.001	(0.25, 0.80)
Exposure to noise (years)	0.297	0.144	2.068	2.067	0.044	(0.008, 0.58)
Constant	−54.583	18.617		−2.931	0.005	(−92.05, −17.11)

B = unstandardized coefficient; β = standardized coefficient; SE = standard error; *p*-value (two-tailed).

**Table A2:** Better-ear hearing threshold model summary.

**R**	**R^2^**	**Adjusted R^2^**	**Std. Error of the Estimate**			
0.624	0.3897	0.349				
F-Statistic, *p*-Value	F = 9.794, *p*-value < 0.001					
**Predictor**	**B**	**SE**	**β**	**t** **-Statistic**	***p***	**95% CI**
Average sound level (dBA)	0.478	0.23	0.245	2.080	0.043	(0.015, 0.94)
Age (years)	0.476	0.16	0.420	2.981	0.005	(0.15, 0.79)
Exposure to noise (years)	0.230	0.166	0.198	1.384	0.173	(−0.104, 0.56)
Constant	−41.413	21.5		−1.926	0.06	(−84.69, 1.86)

B = unstandardized coefficient; β = standardized coefficient; SE = standard error; *p*-value (two-tailed).
